# Improving the Forecasting Accuracy Based on the Lunar Calendar in Modeling Rainfall Levels Using the Bi-LSTM Method through the Grid Search Approach

**DOI:** 10.1155/2023/1863346

**Published:** 2023-12-31

**Authors:** Gumgum Darmawan, Budhi Handoko, Defi Yusti Faidah, Dian Islamiaty

**Affiliations:** Department of Statistics, Faculty of Mathematics and Natural Sciences, Universitas Padjadjaran, Jl.Bandung-Sumedang Km 21 Jatinangor, Sumedang 45363, Indonesia

## Abstract

Rainfall is one of the climatic factors that influence various human activities and affect decision making in daily life activities. High intensity of rainfall can turn into a threat and cause serious problems such as causing various natural disasters. Therefore, it is essential to conduct rainfall forecasting to anticipate and enable preventive actions and can be used as a decision consideration in increasing the productivity and mobility of human activities. The aim of this study is to compare rainfall accuracy between the Gregorian and the lunar calendars using the bidirectional long short-term memory (Bi-LSTM) machine learning model through the grid search approach. This method was used because it can capture patterns arising from the simultaneous effects of two asynchronous calendars, Gregorian and lunar, which were used in this study by finding the right parameters. Monthly rainfall data from Bogor City, Indonesia, were used from the period of 2001 to 2022. The results show that the MAPE of the lunar calendar is relatively smaller at 14.82% which indicates the better forecasting ability than the Gregorian calendar which is 35.12%.

## 1. Introduction

Most rainfall forecasting is based on the Gregorian calendar [1, 2], but many rainfall phenomena are closely linked to the lunar calendar [3]. Earth's climate including variations in rainfall and tides is influenced by the phases of the moon, which are the cornerstone of making the lunar calendar (see [4]). Conversely, rainfall forecasting has generated significant research attention in recent times owing to its complexity and ongoing applications. Hence, methods employing machine learning algorithms in conjunction with time series data are being investigated as viable alternatives to address these limitations (see [5, 6]).

In recent years, machine learning algorithms have been widely employed for time series data predictions, yielding highly accurate results. Machine learning enables the resolution of prediction problems in time series data with a wide range of values. Numerous studies have been conducted on rainfall forecasting using machine learning algorithms, including the support vector machine (SVM) method (see [7, 8]), the deep neural network (DNN) method (see [9, 10]), and the long short-term memory (LSTM) method (see [11–13]).

Bidirectional long short-term memory (Bi-LSTM) is a machine learning method suitable for time series data prediction. It is an extension of LSTM with the capability of retaining dat information from both forward and backward directions. This capability enhances the learning process by offering additional neural networks, leading to more comprehensive results. To obtain the best forecasting model using the Bi-LSTM method, it is essential to determine the optimal parameters for the learning algorithm. Parameter setting and tuning play a significant role in improving forecasting accuracy. One effective approach to determining the optimal parameter setting and tuning is to utilize the grid search algorithm. The grid search algorithm works by systematically combining various parameters used in the model creation process. This method divides the parameter range into a grid and explores different combinations of parameter settings to identify the best parameters for the model. According to [14, 15], the grid search technique improved the model performance.

This study covered three aspects of analysis: (1) rainfall data conversion from the Gregorian-based calendar to the lunar-based calendar, (2) rainfall data modeling and forecasting based on the lunar calendar, and (3) comparison of rainfall forecasting accuracy which is based on the Gregorian calendar and the lunar calendar. The purpose of this study is to forecast the rainfall using the bidirectional long short-term memory (Bi-LSTM) model by the grid search approach. This research is expected to yield an efficient calendar conversion algorithm and can be used as the basis for further research for making an automatization of calendar conversion. Some previous works on the calendar conversion were conducted by [16–21].

## 2. Materials and Methods

### 2.1. Bidirectional Long Short-Term Memory (Bi-LSTM)

LSTM was specifically designed to address the problem of vanishing gradient. LSTM units consist of forget gates, input gates, and output gates, which are used to control the storage or disposal of information. This method has been used in various cases such as sentiment analysis [22], COVID-19 vaccination responses [23], and smartphone data sensors [24]. LSTM usually uses quite complex calculations and high computation in its application. Therefore, this study examines a method with a simpler level of computation but with comparable performance, Bi-LSTM.

Bi-LSTM was proposed by Graves and Schmidhuber to solve a flaw in the recurrent neural network (RNN) and the LSTM model. In both models, information can only be propagated forward, meaning that the time state *t* depends only on the information before time *t* [25]. On the other hand, Bi-LSTM involves two LSTM networks: processing the sequence of data input in the forward direction and processing the sequence of data in the reverse direction (backward). This method can store time series information in two directions and can provide additional training processes. Additional training processes and two-way feature extraction make Bi-LSTM have better performance [26]. In addition, the outputs of the forward and backward LSTM networks are combined on each time sequence.

**Figure 1:**
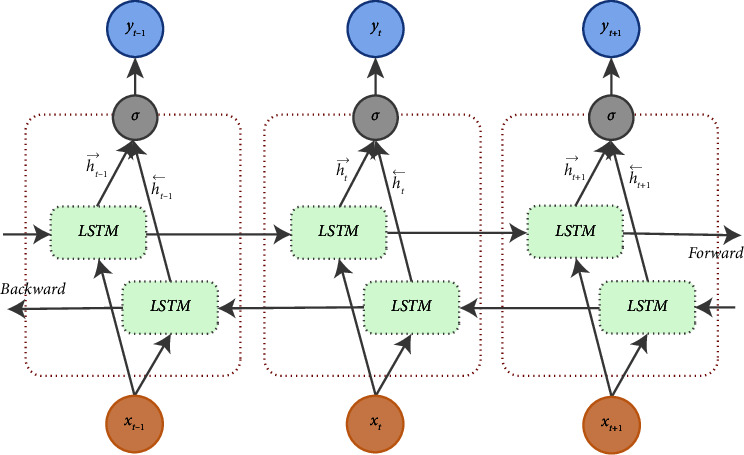
Architecture of bidirectional LSTM (Bi-LSTM).

The Bi-LSTM model can learn past and future information for each input sequence. In addition, Bi-LSTM has two layers of data input that are opposite to each other which enable the model does not forget a long sequence of data information during the training process [27]. Therefore, theoretical prediction performance with Bi-LSTM is better than that with LSTM [28]. The architecture of Bi-LSTM [29] is provided in Figure 1.

Figure 1 shows that the order of the forward layer is the same as in a regular LSTM network that calculates the sequence of *t*  −  1, *t*, and then *t*+1. However, for the backward layer, the hidden layer and output iterated from *t*+1, *t*, to *t*  −  1. $\left(\overset{⟶}{h_{t}}\right)$ and $\left(\overset{⃖}{h_{t}}\right)$ are the forward and backward layers, respectively. According to [30], the process of forward LSTM and backward LSTM can be written as follows:

(1) $$\begin{array}{c} \overset{⟶}{h_{t}}=\text{LSTM}\left(x_{t},h_{t-1}\right),\\ \overset{⟵}{h_{t}}=\text{LSTM}\left(x_{t},h_{t+1}\right). \end{array}$$

It is described in Figure 1 that the hidden layers on each forward and backward are connected and form an output value. The calculation of the output value is shown in the following equation [31]:

(2) $$\begin{array}{c} y_{t}=U_{y}\overset{⟶}{h_{t}}+W_{y}\overset{⟵}{h_{t}}+b_{y}, \end{array}$$

with *y*_*t*_ as the final output value and *U*_*y*_ and *W*_*y*_ as the weight values for *the output gate* on $\overset{⟶}{h_{t}}$ and $\overset{⟵}{h_{t}}$, respectively.

Several studies using the Bi-LSTM method have been conducted by authors in [32] on the case of wastewater flow rate prediction, by authors in [33] on tropical cyclone prediction, by authors in [34] on groundwater content prediction and soil, and by authors in [35] on water content and river water flow prediction.

### 2.2. The Grid Search Method

The grid search is a method used for finding appropriate parameters to improve model performance by trying all combinations of parameters. In its applications, the grid search algorithm is usually combined with cross-validation to form a model evaluation index. The index evaluates model performance by considering data sharing.

In this paper, the grid search algorithm is evaluated by a cross-validation (CV) test. A common form of cross-validation is *k*-fold, which is used to estimate prediction errors in evaluating model performance. It divides datasets into *k* groups of equal size. One of the *k*-fold groups was used as test data while the rest of the groups were used as training data. The parameter pair obtained from the cross-validation test with the smallest error average is the best parameter. This parameter is used in the formation of the model for later testing and evaluation.

### 2.3. Calendar Conversion

**Figure 2:**
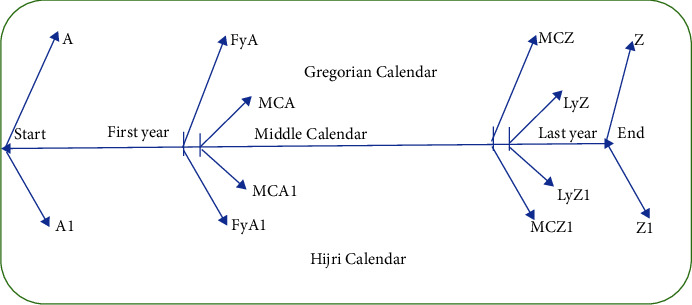
Map of Gregorian to lunar data conversion.

This study utilizes algorithm to convert daily data into monthly data based on the lunar calendar. The month's names and the number of days are referenced from the islamicfinder.com website. The conversion of daily data from the Gregorian calendar to the lunar calendar requires data division into three segments, as illustrated in Figure 2. The conversion process using the “TS” package in R software [36] is done through the following steps:

1. Determine the initial and end dates of the lunar calendar
2. Partition the time interval into three calendar parts
3. Convert boundary points from the Gregorian calendar to the lunar calendar
4. Make these three segments into vector shapes of the dates of the Gregorian and lunar calendars as seen in Figure 2
5. Include calendar attributes, i.e., lunar number and date, e.g., Attr < - c (no, dates)
6. Create a data frame which consists of the combination of the three vectors
7. Input the daily rainfall data based on the Gregorian calendar in a separate column/vector
8. Merge the column vector of daily data with lunar number and dates into the data frame in Step 6
9. Shift the daily data according to the calendar converter and change the name of Gregorian months to the corresponding lunar months

### 2.4. Preprocessing Data

The data preprocessing stage is carried out to improve performance in data processing and prevent errors in the data so that the data used for the prediction process have a high quality. The first stage in the data preprocessing process is data cleaning in which data are adjusted in the presence of missing values. Handling of missing values in data can be done using the mean imputation method, in which missing values are filled up with the average of all known values in a variable.

The second stage is data sharing or data splitting, i.e., data are divided into training and testing data. Training data are used in training models, while testing data are used in evaluation of the selection of model architectures with the best parameters. The total data used in this study were 286 lunar calendar data and 273 Gregorian calendar data. These data were divided into several forecasting lengths: 3, 6, 12, 18, and 24 months.

The third stage is called a scaling or mapping technique which is used to normalize data. The normalization process involves the min-max method. The data normalization process will result in values ranging from 0 to 1. According to [28], the equation used in data normalization is as follows:

(3) $$\begin{array}{c} x_{\mathrm{norm},i}=\frac{x_{i}-x_{\min}\;}{x_{\max}-x_{\min}\;}\;;i=1,2,3,\ldots ,t, \end{array}$$

and the denormalization [37] of the data is

(4) $$\begin{array}{c} x=x^{\prime }\left(x_{\max}-x_{\min}\right)+x_{\min}, \end{array}$$

where *x*_norm_ is a normalised value, *x*_max_ is the maximum value of the entire data, and *x*_min_ is the threshold of the entire data.

### 2.5. Bi-LSTM-Grid Search Modeling

**Figure 3:**
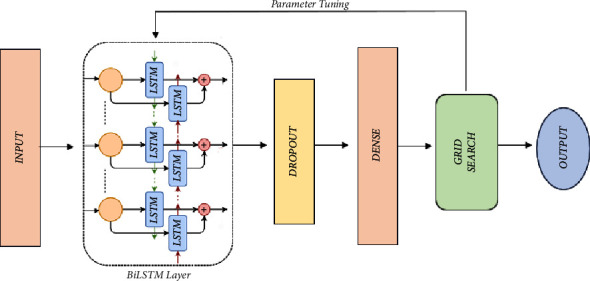
Proposed model Bi-LSTM-grid search.

In building models using the machine learning model such as Bi-LSTM, it is important to select optimal parameters. Parameter determination or tuning parameters are used to control the model so that it can produce better model performance [38]. This study proposed the use of the Bi-LSTM-grid search model (see Figure 3).

Bi-LSTM modeling with grid search consists of input layer, Bi-LSTM layer, dropout layer, dense layer, and the addition of the grid search algorithm to determine the best parameters in the Bi-LSTM learning process and output layer. The input layer is the layer that receives input data, while the Bi-LSTM is a layer in the learning process. Dropout layers are used to prevent overfitting of the model during the learning process. The dense layer is a neural network layer that has functions to convert the output of the previous layer into predicted values. The output layer is a layer that produces outputs or final values in the learning process. The addition of a grid search algorithm is used to determine the best parameters, dividing the range of parameters used into grids and at all points in order to obtain optimal parameters in the learning process of the Bi-LSTM model.

Some of the parameters used in this study consist of one hidden layer, hidden neurons, batch, and epoch. In addition, dropout regulation techniques are also used to avoid overfitting in the model. Adam's optimization or optimizer function is added to determine the optimal weight and reduce errors in the model formation process of maximizing model accuracy. The parameter values set to build the prediction model are given in Table 1.

The determination of neuron numbers in the hidden layer is carried out to obtain the optimal number of hidden neurons. Epoch is a condition where all data have gone through the training process on the network that is formed until it returns to the beginning in one round. Each epoch can be partitioned into batches. Batch is a parameter that determines the sample size used in the process before updating architectural parameters.

The best parameter value from each combination of parameters can be determined using a grid search with the help of cross-validation. It allows for an evaluation of each model with various combinations of predefined parameter limit values. A common form of cross-validation is *k*-fold cross-validation. In this study, the grid search algorithm used 5-fold validation.

## 3. Results and Discussion

### 3.1. Calendar Conversion Results

The data used in this research are daily rainfall data from 1^st^ April 2000 to 31^rd^ December 2022 in Bogor City obtained from the Meteorological, Climatological and Geophysical Agency. The daily rainfall data in the Gregorian calendar were shifted into Gregorian monthly data. The result of the observations of 22 years revealed that the Gregorian calendar has 273 months as seen in Table 2. The results of rainfall data conversion from the Gregorian calendar to the lunar-based calendar can be seen in Table 3 which has 286 months.

### 3.2. Bi-LSTM Prediction Results, Grid Search

The data used in Bi-LSTM modeling were those that passed in the data preprocessing stage, and parameter tuning was conducted using the grid search algorithm. Based on predetermined parameters, 375 models were obtained from a combination of parameters. Of the 375 models, the most optimal combination of parameters was obtained based on the minimum MSE (mean squared error) value for each forecasting length in the Gregorian and the lunar calendars which was continued to the testing process. See Table 4 for the complete results.

Based on the results in Table 4, the smallest MSE was 0.01882 for a 12-month forecasting length with an optimal combination of parameters based on tuning parameters using the grid search such as the number of neurons 20, batch 4, epoch 200, and dropout 0.2. The model with the best combination of parameters obtained from the training data using the grid search was then applied to the testing data. The optimal combination of parameters for rainfall data based on the lunar calendar is shown in Table 5.

According to Table 5, the smallest MSE was 0.01891 with a forecasting length of 3 months, the number of neurons 20, batch 4, epoch 200, and dropout 0.1. Similar to the model in the Gregorian rainfall data, the model with the best combination of parameters obtained from the training data was then applied to the testing data.

The Bi-LSTM model was formed using testing data based on the selection of the best parameters. The forecasting length was evaluated using MAPE based on the lunar and Gregorian calendars which is provided in Table 6.

**Figure 4:**
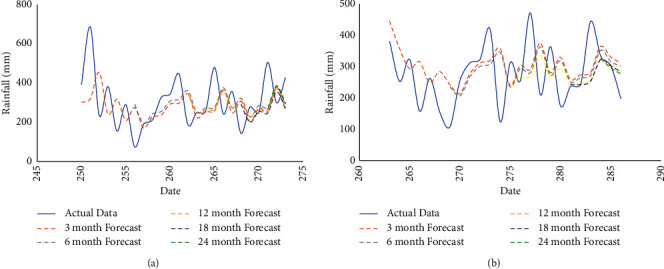
Comparison of actual data and forecasting results of (a) Gregorian-based rainfall data and (b) lunar-based rainfall data.

Table 6 shows the results of model evaluation using MAPE on rainfall data with various forecasting lengths. MAPEs are computed based on the best combination of parameters. The lowest MAPE is the lunar calendar-based rainfall data with a forecasting length of 3 months. The results in Table 6 conclude that the longer the forecasting, the smaller the MAPE. The comparison of actual data and forecasting results of rainfall data based on the Gregorian and lunar calendars using a model with the best parameters for each forecasting length is reported in Figures 4(a) and 4(b).

According to Figures 4(a) and 4(b), the Bi-LSTM model that was formed using the best parameter selected using the grid search algorithm on rainfall data based on the lunar calendar produces predictions that are quite similar to the test data pattern. The MAPE value obtained from rainfall data based on the lunar calendar was relatively lower than the one from the Gregorian. The lowest MAPE was obtained from the lunar calendar-based rainfall data at 14.82% with the best parameter values optimized using the grid search algorithm: the number of neurons 20, batch 4, epoch 200, and dropout 0.1. The criteria of the MAPE value could confirm the accuracy of the model. In conclusion, the best model obtained from the Bi-LSTM grid search was able to provide better results in modeling rainfall data based on the lunar calendar instead of the Gregorian calendar.

## 4. Discussion

Converting the Gregorian-based rainfall data to the lunar calendar gives an advantage in time series analysis. The conversion of daily data to monthly data based on the lunar calendar increases the length of the data series, which provides more information and gives a better forecast. The addition in time series length is an effect of different number of days in a year. In particular, the Gregorian calendar has 365 days, while the lunar calendar has 355 days. In general, the longer the forecasting horizon, the bigger the mean absolute percentage error (MAPE) for both Gregorian and lunar calendars.

The use of lunar calendar-based forecasting for rainfall is because the moon's gravity affects the earth's climate. Furthermore, the moon exerts a more significant influence on earth than the sun due to its closer distance. The moon's position not only affects its phases but also has a gravitational impact on earth's weather. The effect of the moon's gravitational force on earth's rainfall needs further study involving collaboration with astronomers.

Lunar calendars are commonly used in countries with Muslim populations as the majority, such as Indonesia, Saudi Arabia, and the Middle East. The Islamic calendar, known as the Hijri calendar, is derived from the lunar calendar. Religious holidays often affect significant movements of residents who want to visit places of worship or their hometown for family reunions, visit their parents and relatives, and so on. As a result, the lunar calendar has a notable impact on transportation and economy, particularly in countries with large Muslim communities.

The grid search algorithm proposed in this paper remains time-consuming. Each parameter combination takes approximately two hours to complete, which is less efficient. Hence, it is advisable to explore alternative algorithms, such as the genetic algorithm.

## 5. Conclusions

In this paper, we employed the machine learning time series method with the Bi-LSTM model and the grid search approach. The accuracy of forecasting results of rainfall data based on the lunar calendar was evaluated using the mean absolute percentage error (MAPE). We also compare the MAPE of the Gregorian calendar-based rainfall data model and the lunar calendar-based rainfall data model.

The lowest MAPE for the Gregorian calendar-based model was 35.12%, while the lowest MAPE for the lunar calendar-based model was 14.82%, with a forecasting length of 3 months. The smaller MAPE for the lunar calendar-based model suggests a superior forecasting ability compared to the Gregorian calendar-based model. According to the MAPE criteria, the forecasting model based on the lunar calendar can be considered highly accurate. The optimal combination of parameters for rainfall data based on the lunar calendar, as determined through the grid search algorithm, comprises 20 neurons, 200 epochs, a batch size of 4, and a dropout value of 0.1.

## Figures and Tables

**Table 1:** Parameter description.

Parameter	Value
Neuron hidden	5, 10, 15, 20, 25
Batch	4, 16, 32, 64, 128
Epoch	50, 100, 150, 200, 250
Dropout	0,1; 0,2; 0,3
Optimizer	Adam

**Table 2:** Monthly rainfall data (mm) based on the Gregorian calendar.

Year	Gregorian month											
	Jan	Feb	Mar	Apr	May	Jun	Jul	Aug	Sep	Oct	Nov	Dec
2000	—	—	—	449	339	295	376	248	123	222	36	99
2001	219	337	118	581	699	498	368	282	134	70	52	117
2002	376	439	76	635	659	337	339	31	152	186	81	23
2003	47	217	278	138	582	247	266	165	21	0	205	247
2004	291	264	373	292	513	270	355	241	40	74	155	239
2005	188	468	684	417	163	124	163	140	140	207	203	194
2006	264	283	692	446	161	309	132	41	14	6	21	102
2007	160	554	401	395	386	114	7	134	62	167	236	584
2008	311	315	515	516	406	155	63	3	74	160	224	473
2009	254	605	535	385	222	389	128	87	15	64	358	232
2010	417	523	475	84	291	255	138	306	375	427	286	291
2011	389	265	225	219	175	140	36	8	58	284	394	252
2012	384	348	240	318	144	64	41	12	122	260	366	423
2013	851	338	405	344	494	123	275	131	70.1	198.6	259	501.6
2014	1134	623.8	266.7	403.8	219.9	199.1	344.1	249.8	33.6	94.2	548.3	445.7
2015	284.2	345.4	335.7	196.3	148	14.8	0	0	18.8	50.1	457.7	409
2016	272.5	581.7	553.2	461.2	231.2	201.7	252.6	82.6	365.9	386.7	309.6	142.5
2017	293	688.5	283.7	400.8	225.9	130.8	89.1	49.3	33.7	367.4	420.8	320.7
2018	349.1	679.5	448	298.8	140	160.4	25	20.5	161.7	162.4	390.1	251.7
2019	416.3	467.9	234.8	471	207.4	0	51.4	34.8	13.8	196.2	183.8	328.9
2020	421.3	537.1	511.8	344.2	407.6	96.3	95.1	62	100.4	292.6	165.7	343.9
2021	392	687.1	235.4	382.2	155.3	289.6	74.3	189.7	211.6	327	343.8	446
2022	188.2	250	255.3	479.6	242.3	356.3	143.4	278.6	250.7	505.3	299.2	428.7

**Table 3:** Conversion of monthly rainfall (mm) data based on the lunar calendar.

Year	Lunar month^*∗*^											
	Muh	Saf	Raw	Rak	Jaw	Jak	Raj	Syb	Ram	Syw	Dzq	Dzh
1420	—	—	—	—	—	—	—	—	48	493	263	348
1421	329	190	127	218	33	101	210	336	119	475	790	363
1422	482	253	202	54	68	92	204	498	169	239	741	488
1423	320	259	97	71	179	78	23	70	208	283	119	582
1424	247	197	234	17	4	165	286	246	220	287	360	426
1425	405	189	386	120	67	10	99	200	140	171	499	699
1426	365	167	187	187	140	157	154	243	197	241	270	685
1427	461	146	309	132	41	5	15	6	62	149	332	401
1428	1137	408	262	253	100	94	47	102	64	289	227	649
1429	313	500	406	373	120	63	3	113	149	196	473	250
1430	520	612	371	219	399	145	18	82	65	214	315	245
1431	351	523	456	269	198	321	100	185	395	282	375	378
1432	199	409	264	184	231	157	157	13	3	60	201	456
1433	203	347	353	293	332	123	119	43	12	73	170	236
1434	490	506	714	385	340	270	438	135	314	64	102.6	166.1
1435	270.4	495.2	1128	624.1	266.4	394.9	227.9	200	343.2	171	112.9	47.6
1436	517.2	196.9	449.8	404	328.7	223.3	230.6	14.9	0	0	0	55.5
1437	142.7	479.1	354.3	342.7	638.6	466.2	373.6	183.6	258.8	194.7	90.1	358.3
1438	348.7	346.4	134.1	259.5	682.8	318.2	357.7	262.2	91.8	80	101.7	19.7
1439	262.4	370.6	365.1	266.8	684.4	485.8	380.5	109.4	131	159.7	24.8	89.8
1440	94.6	198	410.8	248.8	400.2	468.3	209.5	461	202.8	77.9	51.4	20.8
1441	27.8	190.5	167.9	252.3	383.4	536.9	441.6	449.9	264	288.5	135.7	72.5
1442	35.2	139.5	294.9	303.4	253.5	656.7	381.3	253.2	323.1	158	263.1	154.6
1443	108.8	256.7	312.6	323.9	419.2	124.9	313.3	255.3	471.6	210.6	363.9	175.5
1444	237.6	244.1	444.7	334.1	303.4	198.6	—	—	—	—	—	—

^*∗*^The names of lunar month are Muh (Muharram), Saf (Safar), Raw (Rabi'ul Awal), Rak (Rabi'ul Akhir), Jaw (Jumadil Awal), Jak (Jumadil Akhir), Raj (Rajab), Syb (Sya'ban), Dzq (Dzul qo'dah), and Dzh (Dzul hijjah).

**Table 4:** Results of Bi-LSTM-grid search tuning parameters on the Gregorian calendar.

Forecasting length	Parameter				MSE
	Neuron	Batch	Epoch	Dropout	
3	20	4	200	0.1	0.01926
6	20	4	200	0.1	0.01945
12	20	4	200	0.2	0.01882
18	15	4	200	0.1	0.02004
24	20	4	200	0.1	0.01964

**Table 5:** Bi-LSTM-grid search tuning parameter results on the lunar calendar.

Forecasting length	Parameter				MSE
	Neuron	Batch	Epoch	Dropout	
3	20	4	200	0.1	0.01891
6	15	4	200	0.3	0.01907
12	20	4	200	0.3	0.01896
18	20	4	200	0.2	0.01933
24	15	4	200	0.1	0.01926

**Table 6:** MAPE value in the Gregorian and lunar calendars.

Forecasting length	MAPE	
	Gregorian (%)	Lunar (%)
3	35.12	14.82
6	40.27	15.46
12	37.29	26.81
18	47.36	40.76
24	49.14	42.65

## Data Availability

The data used to support the findings of this study are available from the corresponding author upon request.
